# DEVELOPMENTAL DYSPLASIA OF THE HIP: DO THE RESPONSIBLE FOR SCREENING KNOW WHAT TO DO?

**DOI:** 10.1590/1413-785220162406165319

**Published:** 2016

**Authors:** Bruno Gonçalves Schröder e Souza, Tiago Evangelista de Melo, Thiago Mattos Resende, Rafaela Clara Resende da Silva, Soraya Amanda Cruz, Valdeci Manoel de Oliveira

**Affiliations:** 1. Faculdade de Ciências Médicas e da Saúde de Juiz de Fora (SUPREMA), Juiz de Fora, MG, Brazil.; 2. Hospital e Maternidade Terezinha de Jesus, Orthopedics and Traumatology Service, Juiz de Fora, MG, Brazil.

**Keywords:** Hip dislocation, congenital. Risk factors. Education, continuing. Prognosis

## Abstract

**Objective:**

To evaluate the knowledge on developmental dysplasia of the hip (DDH) by professionals involved in its diagnosis.

**Methods:**

This is a cross-sectional study using questionnaires to assess the knowledge about DDH. Orthopedic surgeons and pediatricians, residents and medical students from a tertiary teaching hospital were included in the study.

**Results:**

We evaluated 142 medical students, eight orthopedic residents, ten pediatric residents, seven pediatricians, and nine orthopedic surgeons; 50% declared not having examined any DDH case in the last year and only three had diagnosed more than 10 cases during their career. Regarding self-assessed knowledge (0-10), the average score was 4.25 [n=186; SD=2.43]. Nineteen percent of the participants ignored semiological tests and 26.1% of pediatricians (specialists and residents), were unaware of how to perform them. The most acknowledged and neglected risk factor was pelvic presentation (68%) and CMT (9.3%), respectively. None of the participants were able to identify all the risk factors. The average number of risk factors identified was two (n=186; SD=1.58). Forty seven point three percent of the participants failed to recognize the time of birth as the ideal moment for diagnosis; 17% reported it was after the first month. Regarding neglected severe DDH, 45.3% failed to recognize its natural history.

**Conclusion:**

Knowledge on DDH among health professionals who are involved in screening is flawed. Level of Evidence IV, Developing a Decision Model.

## INTRODUCTION

Developmental dysplasia of the hip (DDH) is described a set of abnormalities ranging from abnormally acetabulum (dysplasia) and mild subluxation of the femoral head to fixed displacement (congenital dislocation).^1^ DDH is a multifactorial disease, which risk factors include: female gender, family history, and factors that decrease the potential of fetal movements, such as multiple pregnancy, oligohydramnios, primiparity and high weight at birth. Other congenital deformities which affect the cardiovascular and genitourinary systems and the locomotor system, such as congenital muscular torticollis and crooked foot, may also be related.^2^ It is estimated that the incidence of DDH is 1.5-20/1,000 live births, and it is 4-8 times more prevalent in women.^1^ ^,^ ^3^ Early detection of this condition facilitates the use of minimally-invasive procedures that are generally more effective.^4^

Diagnosis of DDH is dependent upon a high level of clinical suspicion and physical examination^5^. Diagnosis of DDH by examination is positive when either the Ortolani or Barlow sign is positive. Other clinical signs may be detected at various stages of the disease development.^6^ In the presence of risk factors, the physician should increase the level of suspicion and additional tests may be required.^2^ Ultrasonography is the preferred method in these cases. In most hospitals, these methods are only used in cases with confirmed risk factors or changes in physical examination.^6^

The optimum time for diagnosis is right after birth, during the first physical examination of the child by the pediatrician. Delays in diagnosis can be catastrophic, since the absence of the femoral head centered in the acetabulum may affect the child's development, typically leading to a progressive worsening of the disease. Early diagnosis results in a 95% success rate of clinical treatment with a low risk of complications.^6^ In the absence or delay of a diagnosis, treatment is more complex, increasing the risk of morbidity and decreasing the probability of normal hip development.^1^ ^,^ ^3^ ^,^ ^7^ This is reflected by the requirement for increasingly invasive procedures to treat the disease as the child grows.

Therefore, in this particular condition, a correct history, identification of risk factors and systematic clinical examination are crucial for the diagnosis and decision-making during follow-up. The aim of the present study was to evaluate and analyze the level of knowledge on DDH among medical students, orthopedics and pediatrics residents, orthopedic surgeons and pediatricians from a tertiary philanthropic teaching hospital exclusively for public service.

## MATERIAIS E MÉTODOS

**Figure 1:**
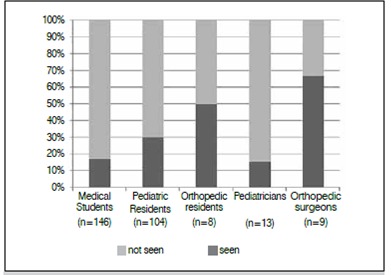
Frequency of patients with suspected DDH in the previous year.

The present cross-sectional study involving 186 subjects (13 pediatricians, nine orthopedists, 10 pediatric residents, eight orthopedics residents, and 146 fifth and sixth year medical students of both genders) was conducted between October 2013 and March 2014. This study was approved by the Research Ethics Committee of our institution (approval number CAAE 18393013.6.0000.5103), and all participants signed an informed consent form. There were no exclusions. Among the 254 individuals included by the researchers, 68 refused to participate. To assess the level of knowledge of health professionals on DDH, a questionnaire especially developed for this purpose was used by the researchers, based on previous similar research and grounded in the current medical literature. (Figure 1) Results are presented as descriptive statistics, which were tabulated, analyzed in Microsoft Excel 2013 and compared using GraphInstat 3.0 software using the chi-square test and analysis of variance for dichotomous and continuous variables, respectively. We adopted *p* < 0.05 as significant.

## RESULTADOS 

**Figure 2:**
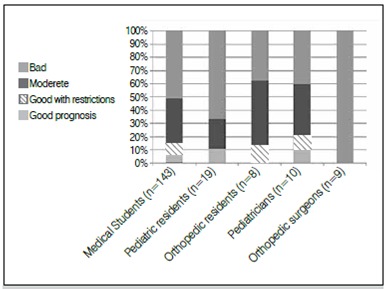
DDH prognostic evaluation regarding untreated DDH patients.

In the present sample population, 81.1% (151/186) of participants reported never having made a diagnosis of DDH in their professional or academic life. Furthermore, only one orthopedist reported having diagnosed more than ten cases in his career. Figure 2 lists the frequency with which each group has encountered at least one patient with suspected DDH in the previous year (independent variables were *p* = 0.0005 (chi-square test), when orthopedists and pediatricians were associated with their respective residents). 

**Figure 3:**
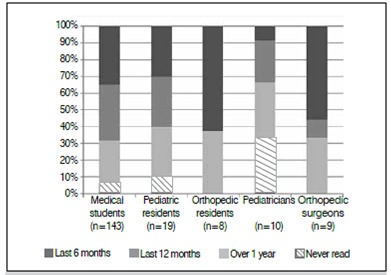
When did the professional read for the last time literature on DDH.

Regarding the prognosis of severe DDH, when it has not been treated during the early stages of life, all orthopedists correctly indicated that there is a poor prognosis associated with multiple surgeries throughout life. A significant portion of pediatricians recognized the situation as moderately severe, and, worryingly, 15.7% of pediatric residents and pediatricians reported that the prognosis was good. Figure 3 presents the distribution of answers by specialty.

**Figure 4:**
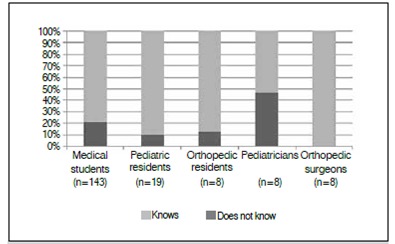
Knowledge on Ortolani and Barlow's maneuvers.

**Figure 5:**
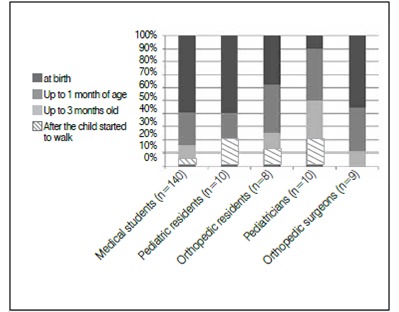
Age perceived as optimal for the diagnosis of DDH.

When asked to report when they last read about the subject, 7.5% of participants reported never having read about it. Among pediatricians, this value was 33.3%. Figure 4 shows the distribution by specialty. Regarding the maneuvers during physical examination, 20.9% of respondents were unaware of them. Figure 5 presents the distribution by specialty.

None of the respondents correctly identified all eight potential risk factors presented. The average number of correctly identified risk factors was two (standard deviation = 1.58). Risk factors most frequently identified were breech presentation (65%), gender (43%), and family history (33%). The most overlooked factor was congenital muscular torticollis (9.7%). Table 1 shows the relative frequency of correctly identified risk factors per specialty.

**Table 1:** Relative frequency of DDH risk factors by specialty

	Breech presentation	Gender	Family history	Gestational diabetes	Oligohydramnios	Nulliparity	Congenital anomalies	TMC
Medical Students (n=143)	65.5%	47.2%	31.7%	28.9%	22.5%	14.8%	9.9%	7.7%
Pediatric residents (n=19)	80.0%	30.0%	40.0%	10.0%	30.0%	50.0%	20.0%	10.0%
Orthopedic residents (n=8)	87.5%	37.5%	25.0%	62.0%	62.5%	12.5%	12.5%	25.0%
Pediatricians (n=10)	46.2%	30.8%	46.2%	30.8%	30.8%	7.7%	7.7%	7.7%
Orthopedic surgeons (n=9)	88.9%	33.3%	44.4%	55.6%	33.3%	22.2%	11.1%	33.3%
Total	65.6%	43.0%	32.8%	29.6%	25.3%	16.1%	10.2%	9.7%

**Figure 6:**
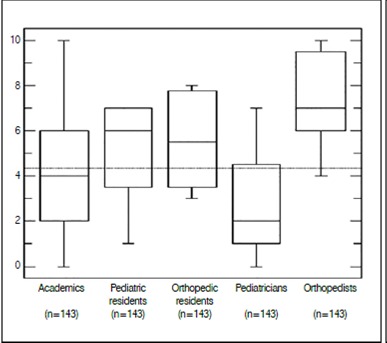
Box -plot of the self- assigned grades on the knowledge on DDH in relation to the average line.

When asked to indicate the best age to make the diagnosis, 6.4% (12/186) reported that it was appropriate to do so after the infant had started to walk, and only 52.6% (98/186) identified birth as the ideal time for the diagnosis of DDH. Figure 6 shows the frequency of correct answers by specialty.

Finally, when asked to assess their own knowledge of the subject, respondents were attributed an average score of 4.25±2.43 (in a scale 0-10). Figure 7 presents the significant differences between the specialties (*p* < 0.0001). We observed a weak linear correlation between the self-assigned score and the number of correctly identified risk factors (y = 0.0874x + 1.9831, r^2^=0.018).

## DISCUSSÃO

DDH has a prevalence of 1.6-28.5 cases per thousand live births. Several strategies for neonatal screening of this disease have been developed, since a lack of a diagnosis may lead to an increased severity, costs and complexity of the procedures required for treatment.^8^ Clinical trial has traditionally been the standard screening test in most countries where general practitioners, obstetricians and pediatricians are responsible for this initial approach. Although considered cost effective by Lehmann HP et al.,^8^ they also highlighted that this approach requires constant training and updating of the professionals involved, in order to ensure that these clinicians correctly identify the risk factors of DDH and are empowered to carry out the diagnostic techniques.^8^ ^,^ ^9^

The present study showed that professionals who perform the first examination may experience great difficulty in achieving the clinical maneuvers and identifying risk factors. Uzel et al.^10^ reviewed the medical knowledge on DDH before and after a lecture and found that 18.6% of participants did not recognize DDH as a treatable disease with controllable consequences. In our study, 12.8% of pediatricians and pediatric residents believed that the prognosis of severe DDH which has not been treated in the early stages of life was good, with minimal sequelae and did not require any surgery. Similarly, 20.9% of respondents were unaware of the physical examination maneuvers for diagnosis of DDH. Uzel et al.^10^ concluded that the knowledge and attitudes of physicians towards DDH must be improved by promoting continuing education programs.

It has previously been reported that the natural evolution of hip instability may be spontaneously resolved.^3^ In the same study, it was demonstrated that indiscriminate treatment of the disease increased the incidence of unnecessary complications, such as compression of the femoral nerve and necrosis of the femoral head. These findings appear to have been widely disseminated to pediatricians. On the other hand, the incidence of these complications are rare following the concentric reduction of the hip.^11^ One study estimated that the incidence of necrosis in cases treated prior to six months is 2.5/1,000 children whom were referred to a specialist, whereas after six months the rate was 109/1,000.^8^ Moreover, delayed treatment of DDH is associated with greater and more frequent complications and morbidity associated with the need for invasive surgical procedures, such as open cuts, pelvic osteotomies and femoral osteotomies.^12^ The prognosis of undiagnosed DDH is extremely unfavorable; permanent lameness and multiple invasive surgical procedures and coxarthrosis (hip arthritis) before 40 years-of-age are common in these patients. Sakellariou et al.^13^ demonstrated that DDH is the main cause of total hip arthroplasty in young adults, and the socioeconomic implications of this disease are potentially devastating. Furthermore, it is recognized that hip arthroplasties performed before the age of 50 are associated with a higher release rate and early review. These findings suggest that there appears to be a discrepancy in the understanding of pediatricians and orthopedists about the condition, which may explain the less than emphatic approach to effective screening by the former.

Worldwide, there are several approaches to screening and treatment. In some European countries, screening with quantitative ultrasound using the Graf method, which may reduce the incidence of delayed diagnosis, has become a public health policy.^5^ In Austria, following the implementation of a national screening program for DDH with the widespread use of ultrasound, the annual incidence of open reduction surgery fell from 3.5/1,000 to 0.16/1,000 live births over a period of 16 years, and pelvic osteotomies and acetabuloplasty decreased by 46%. Hospital readmissions for treatment of DDH decreased from 9.5 to 3.6/1,000 live births.^14^ A decrease in the overall cost related to this disease was estimated to be 1/3 in a decade. On the other hand, a high rate of false positive results and the increased cost are potential disadvantages of the indiscriminate use of complementary tests.^15^ ^-^ ^17^ In addition, cost effectiveness studies have failed to demonstrate the superiority of global screening when compared to selective screening via ultrasound of suspected or high risk cases.^18^ However, the meta-analyzes of DDH screening have not included any studies that examine the effect of screening associated with early treatment versus no screening associated with delayed treatment. In a recent review, open reduction surgery rates, which can be considered as screening failure was 0.28/1,000 live births (0:15-12:41/1,000) on average, in studies with ultrasound screening of all newborns; 0.7/1,000 (0.56-0.87/1,000) in studies with selective screening; and 1.28/1,000 on average in studies with clinical screening (0.29-3/1,000).^14^ This reinforces the requirement for some kind of effective screening. ^5^

A meta-analysis by Hundt et al.^19^ demonstrated that children born with pelvic presentation, female babies, children with positive family history and those who were Ortolani-positive in clinical examination have an increased risk for DDH. Fitch^20^ has previously outlined that there is a higher risk for the development of DDH if the disease is present in a first-degree relative, the fetus has breech presentation, the newborn is habitually wrapped in swaddling clothes, or if congenital deformity calcaneal valgus foot is detected. In addition, Fitch^20^ noted that clinical screening alone sometimes results in late diagnosis, and the presence of risk factors may trigger the selective use of ultrasound exam. This strategy is adopted around the world and seems to be a logical and suitable approach, particularly in places where knowledge regarding DDH is poor, as observed in the present study.

### Study limitations

The present study had several limitations. Firstly, the generalization of our results is limited due to the selection bias that exists in a convenience sample. Particularly, as the present study evaluated the situation in a teaching hospital, the knowledge on DDH in samples from other scenarios may be even worse. Nevertheless, since it is a service responsible for the birth of one third of all children in a medium-sized city in the Southeast, where more than one million people reside, it seems appropriate to disclose the results of our survey as the national literature does not contain any similar data. Secondly, the comparative analysis of the data was impaired in some cases due to the small number of professionals in each category. When necessary, we grouped the data to perform statistical analysis. Finally, a questionnaire was developed to obtain the survey data. Although this enabled flexibility in the registration of data of interest, this method is not validated, and thus, may be difficult to compare to other studies and ensure internal validation of our research. 

## CONCLUSION

Knowledge on DDH among professionals directly involved in treatment of this condition is flawed. Special attention should be paid to the continuing education of pediatricians who are directly responsible for screening patients. The completion of the present work leading to a thesis was only possible due to the collaboration of a group of people, to whom I would like to extend my heartfelt thanks.
